# Europe’s hepatitis challenge

**DOI:** 10.2471/BLT.18.021218

**Published:** 2018-12-01

## Abstract

A hidden epidemic is coming to light in Europe but under-reporting and late diagnosis continue to hamper progress in preventing hepatitis B and C infections. Vijay Shankar Balakrishnan reports.

Most people living with chronic hepatitis B and C are unaware of their infection until the virus has caused serious liver damage.

“I treat about 15 patients a day,” says Dr Ubaldo Visco Comandini, a clinical virologist at the National Institute for Infectious Diseases in Rome, Italy. “About 70% of them have viral hepatitis.”

Many of Comandini’s patients come to him with late-stage complications of chronic hepatitis B or C infections, such as cirrhosis and hepatocellular carcinoma.

Had they been diagnosed and treated earlier, these severe, often fatal, complications could have been prevented.

Hepatitis B and C infections are responsible for some 96% of all hepatitis deaths globally. They are a major focus of the 2016 World Health Organization (WHO) Global Health Sector Strategy on viral hepatitis, which calls for the elimination of viral hepatitis as a major public health threat by 2030 (i.e. a 90% reduction in annual new infections and a 65% decrease in annual viral-hepatitis-related deaths).

Europe is one of the regions struggling to combat the viral hepatitis epidemics, but the exact size of the problem remains unclear, partly because of the asymptomatic nature of hepatitis infection.

“Many people with hepatitis B and C infections show no obvious symptoms, so the disease often goes undiagnosed,” says Erika Duffell, an epidemiologist at the European Centre for Disease Prevention and Control (ECDC) in Stockholm, and an author of *Hepatitis B/C in the countries of the European Union/European Economic Area (EU/EEA): a systematic review of the prevalence among at-risk groups,* published in February in *BMC infectious diseases*.

The study is partly based on a 2016 ECDC survey, which found that 45–85% of hepatitis B infections, and 20–89% of hepatitis C infections in European countries go undiagnosed for years.

A key public health objective, therefore, is to ensure that such infections are diagnosed earlier.

Just as voluntary testing for HIV status has become an important strategy for the HIV epidemic, people are encouraged to check their hepatitis status through testing.

Hepatitis B and C infections can be treated with medicines that slow the progression of cirrhosis and reduce the incidence of liver cancer. With new medicines, hepatitis C can be cured. So, the earlier such infections are detected, the less likely patients are to transmit their infection to others and the more likely that their treatment outcomes will be positive.

To prevent infection in the first place, many European countries now include the hepatitis B vaccine in their national immunization schedules and, as a result, most of these countries report less than 1% prevalence.

“To eliminate hepatitis C virus infection as a public health threat in Europe, we must focus on people who inject drugs.”

Dagmar Hedrich

In Italy, hepatitis B prevalence stands at an estimated 0.7%, a level which Anna Rosa Garbuglia, a researcher and virologist at the National Institute for Infectious Diseases, credits to the country’s hepatitis B vaccination campaign which began in 1991.

“The decline in hepatitis B is greatest in countries with high vaccination coverage, that is to say, equal or above 95%,” says Duffell, underlining the importance of comprehensive vaccination programmes for hepatitis B. The hepatitis B virus is transmitted through exposure to infective blood, semen and other body fluids (the virus can be transmitted from mother to infant during delivery, and from adults to children through household transmission).

There is currently no vaccine for hepatitis C, and infection prevention is complicated by the populations most affected.

Hepatitis C is mostly transmitted through exposure to infective blood and is the most common infectious disease in people who inject drugs, with transmission occurring through the sharing of syringes, needles and other injecting equipment.

According to the ECDC review, injection of drugs accounts for 77.6% of hepatitis C transmission, while 11 of the 16 European countries with recent data report national hepatitis C prevalence estimates in this group of more than 40%.

 “To eliminate hepatitis C virus infection as a public health threat in Europe, we must focus on people who inject drugs,” says Dagmar Hedrich, lead scientist for harm reduction at the European Monitoring Centre for Drugs and Drug Addiction, an EU agency based in Lisbon, Portugal.

Duffell agrees, noting that efforts to date have been inadequate in many European countries, particularly with regard to comprehensive harm reduction interventions, such as those combining needle and syringe programmes and opioid substitution treatment. However, people who inject drugs are not the only ones in the population at risk of hepatitis C infection. There are reports of increasing incidence of hepatitis infection among men who have sex with men and among prisoners and migrants.

To meet the needs of these different population groups, Hedrich believes that all governments need a national hepatitis C virus policy. Ideally, such a policy ensures access to testing for blood-borne viruses and other sexually transmitted infections, and greater accessibility to effective treatment in the form of direct-acting antiviral medicines that are recommended in the WHO hepatitis C guidelines.

“There is a need to focus on the overall health of drug users, including through integrated HIV–HCV services,” says Hedrich, adding that these services must be tailored and accessible for people who inject drugs.

Some countries are already providing integrated services for injecting drug users. For example, Germany launched a strategy that integrates care for HIV, hepatitis B and C and other sexually transmitted infections in 2016 and became one of the first European countries to guarantee universal access to treatment of chronic hepatitis C.

“Germany offers a good example of leadership in viral hepatitis response,” says Dr Antons Mozalevskis, Medical Officer for hepatitis at the WHO Regional Office for Europe in Copenhagen, Denmark.

Under Italy’s National Programme for Viral Hepatitis Prevention, screening is now provided free of charge. In addition, the health ministry and the Italian Medicines Agency are making treatment for hepatitis C widely available: “Since April 2017, direct-acting antivirals are provided at no cost to Italian residents infected with hepatitis C,” Condamini says.

Romania, which, reportedly, has high prevalence of both hepatitis B and C, is developing a comprehensive hepatitis action plan aligned with the WHO Global Health Sector Strategy on Viral Hepatitis 2016–2021 and the European Action Plan.

“Unless countries have robust epidemiological data information, they cannot know the true scale of the viral hepatitis epidemics.”

Erika Duffell

Duffell argues that further progress will depend on the extent to which countries are able to shed light on the true extent of the epidemic. For this reason she is calling for a standardized EU prevalence hepatitis survey to provide the kind of strategic information policy-makers and public health authorities need to develop, implement and monitor more effective responses.

“Whilst some EU/EEA countries have already made considerable progress on the implementation of primary and secondary prevention measures, only a few countries have developed monitoring programmes,” Duffell says. “Unless countries have robust epidemiological data information, they cannot know the true scale of the viral hepatitis epidemics and will be unable to make progress towards their goals of elimination.”

Those goals are set out in the 2016 WHO Action plan for the health sector response to viral hepatitis in the WHO European Region, which addresses all five hepatitis viruses, but focuses mainly on Hepatitis B and C. The main goal of the plan is the elimination of viral hepatitis as a public health threat in the European Region by 2030 through the reduction of transmission, morbidity and mortality due to viral hepatitis and its complications, and by ensuring equitable access to comprehensive prevention, and recommended testing, care and treatment services for all.

**Figure:**
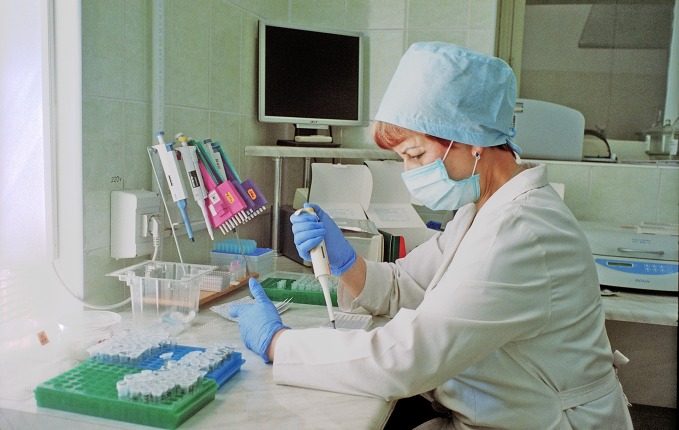
Testing for hepatitis in Ukraine

MSF/Aleksandr Glyadyelov

**Figure:**
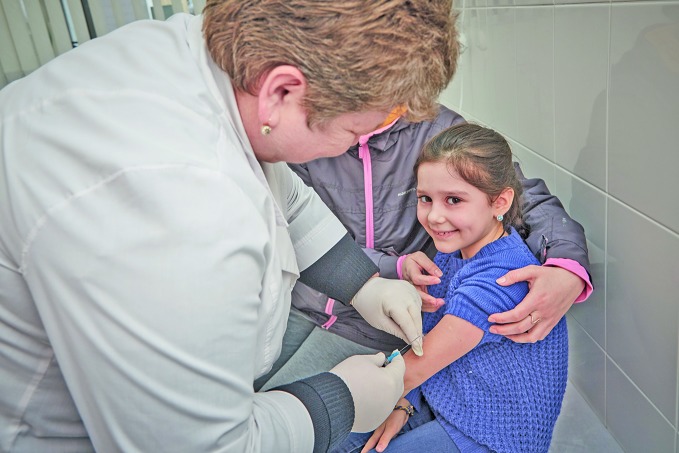
A girl receives the hepatitis B vaccine at a medical facility in Ivano-Frankivsk in Ukraine.

UNICEF/P. Zmey

